# Nontuberculous Mycobacteria–associated Lung Disease in Hospitalized Persons, United States, 1998–2005

**DOI:** 10.3201/eid1510.090196

**Published:** 2009-10

**Authors:** Megan E. Billinger, Kenneth N. Olivier, Cecile Viboud, Ruben Montes de Oca, Claudia Steiner, Steven M. Holland, D. Rebecca Prevots

**Affiliations:** George Washington University, Washington, DC, USA (M.E. Billinger); National Institutes of Health, Bethesda, Maryland, USA (K.N. Olivier, C. Viboud, R. Montes de Oca, S.M. Holland, D.R. Prevots); Agency for Healthcare Research and Quality, Rockville, Maryland, USA (C. Steiner)

**Keywords:** Mycobacteria, atypical, nontuberculous mycobacteria, tuberculosis and other mycobacteria, prevalence, hospitalizations, United States, research

## Abstract

The prevalence and trends of pulmonary nontuberculous mycobacteria (NTM)–associated hospitalizations in the United States were estimated using national hospital discharge data. Records were extracted for all persons with a pulmonary NTM International Classification of Diseases code (031.0) hospitalized in the 11 states with continuous data available from 1998 through 2005. Prevalence was calculated using US census data. Pulmonary NTM hospitalizations (031.0) increased significantly with age among both sexes: relative prevalence for persons 70–79 years of age compared with those 40–49 years of age was 15/100,000 for women (9.4 vs. 0.6) and 9/100,000 for men (7.6 vs. 0.83). Annual prevalence increased significantly among men and women in Florida (3.2%/year and 6.5%/year, respectively) and among women in New York (4.6%/year) with no significant changes in California. The prevalence of pulmonary NTM–associated hospitalizations is increasing in selected geographic areas of the United States.

## Abstract 2

This bacterium is an underappreciated cause of lung disease and infection rates appear to be increasing.

Clinic- and laboratory-based studies since the 1980s have shown an increased prevalence of persons with nontuberculous mycobacterial (NTM) pulmonary disease (*1**,**2*) with a predominance of women >60 years of age who have no underlying risk factors (*3**–**5*). NTM comprise a multispecies group of environmental organisms living in soil as well as in treated and untreated water sources. These mycobacteria were first identified as human pathogens in the 1950s when 1%–2% of patients in tuberculosis (TB) sanitaria did not respond to traditional TB treatment. Their illnesses were caused by organisms that were not *Mycobacterium tuberculosis*. These patients tended to be older than those having TB, were more likely to be white, and to have underlying lung disease (*6**,**7*).

The success of TB elimination efforts has resulted in a continued decline in the incidence and prevalence of tuberculosis in the United States. In 2007, the incidence of TB in the United States was 4.4/100,000 population, and 2.1/100,000 among US-born persons, the lowest rates since reporting began in 1953 (*8*). The apparent increase in NTM disease has occurred during the same period that TB has been declining. Although NTM are not transmissible, the diseases they cause may greatly affect public health and medical care resources. In some state health departments, findings of an acid-fast bacilli, indicative of mycobacteria, are reportable (*9*), and may trigger a public health investigation with substantial expenditure of resources until species identification is confirmed.

Population-based surveys conducted during 1981–1983 estimated the prevalence of pulmonary NTM disease at 1–2 cases/100,000 persons in the United States (*10*). A more recent retrospective analysis from Ontario, Canada found an average annual increase of 8.4% for the isolation prevalence of NTM at the Ministry of Health Mycobacterial Laboratory between 1997 and 2003 (*11*). Similar trends have been noted in other areas of the world (*12**–**16*). However, no current US nationally representative data exist regarding the prevalence of pulmonary disease associated with NTM. Furthermore, information is limited regarding risk factors associated with the disease. Our study describes the prevalence, demographic characteristics, and trends of pulmonary NTM–associated hospitalizations during 1998–2005.

## Methods

### Data Source and Study Population

We used data from the Agency for Healthcare Research and Quality’s Healthcare Cost and Utilization Project (HCUP), specifically the State Inpatient Databases (SID). The SIDs provide record-level data, without personal identifiers, on nearly 100% of community hospital discharges in participating states. Records were included for hospitalizations that had an International Classification of Diseases, 9th Revision, Clinical Modification (ICD-9-CM), code associated with pulmonary NTM (031.0) as a primary or secondary discharge diagnosis. The study population included all records for persons hospitalized with pulmonary NTM as a primary or secondary diagnosis in the 11 states participating in HCUP (Arizona, California, Colorado, Florida, Illinois, Iowa, Massachusetts, New Jersey, New York, Washington, and Wisconsin) during the years specified (*17*). These states represented 42% of the US population during the study period.

### Data Analysis

Data elements available in the HCUP dataset included year of hospitalization, age when hospitalized, sex, state where hospitalization occurred, type of NTM infection (pulmonary, disseminated, cutaneous, unspecified, or other) and up to 29 possible secondary diagnoses. No information on mycobacterial species is available in this dataset. Because NTM is known to be a common opportunistic infection among people with AIDS, particularly before the widespread availability of combination antiretroviral medications (*18*), we limited our analysis to non-AIDS NTM using the code for HIV/AIDS (042), which indicates hospitalizations where AIDS was known to be an underlying illness. Additionally, we restricted our analysis to the 1998–2005 study period to avoid misclassification among types of NTM because the ICD-9-CM code for disseminated NTM was introduced in 1997. Before implementation, hospitalizations associated with disseminated NTM may have been included in the 4 other NTM categories (pulmonary, cutaneous, unspecified, other). We examined prevalence trends in pulmonary NTM by age and sex and described the most frequently associated underlying illnesses. To analyze the most frequent secondary underlying illnesses, we grouped the following conditions/codes as chronic obstructive pulmonary disease (COPD): obstructive chronic bronchitis with and without exacerbation (ICD-9-CM 491.21, 491.22); emphysema not elsewhere classified (492.8); chronic obstructive asthma (493.20); and chronic airway obstruction not elsewhere classified (496).

To estimate prevalence of hospitalizations, we used age- and sex-specific US census data for participating states during the study period; both individual years and midpoint population (average of 2001–2002 census population estimates) were used as appropriate. Although prevalence more often refers to the number of persons with a condition in a population at a determined time, we use it here to describe the number of hospitalizations among persons with NTM. To compare prevalence among states, we calculated age-and sex-adjusted rates using the US census 2000 reference population; χ^2^ tests were used to determine significance among groups at a significance level of p<0.05. Data analyses were calculated using SAS 8.0 and 9.1 (SAS, Cary, NC, USA) and EpiInfo version 3.4 (Centers for Disease Control and Prevention, Atlanta, GA, USA). The average annual percent increase in prevalence and the significance of these trends were estimated by use of Poisson regression models. Prevalence was modeled as a function of time, with prevalence as the dependent variable and time as the independent variable; Pearson’s scale factor was used to account for overdispersion. Model fit was assessed by the value of the scaled Pearson χ^2^, which equals the value divided by the degrees of freedom (value/DF); a value of 1 indicates that the model is a good fit. Wald 95% confidence limits were estimated as well. For modeling trends by age and sex for all 11 states combined, separate models were fit for each age and sex group. Prevalence was defined as the number of observed cases in a given age and sex group for each year as the numerator and the estimated annual population for the specified age and sex group for that year as a denominator, modeled in SAS as the observed count data with a log population offset. For estimation of average annual percent change for men and women in 3 states (California, Florida, and New York), age-adjusted prevalence was the dependent variable, modeled as expected number of cases with a log population offset; time (year) was the independent variable. Models were fit separately for men and women. A constant term was included as part of these equations.

## Results

**Figure 1:**
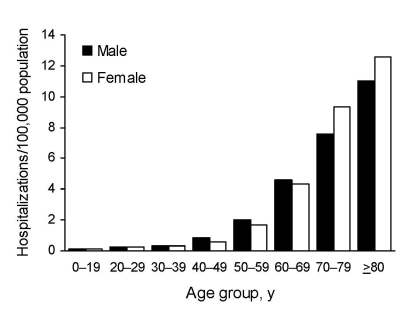
Average annual prevalence of non-AIDS pulmonary nontuberculous mycobacteria–associated hospitalizations by age group and sex, Healthcare Cost and Utilization Project state inpatient databases, USA, 1998–2005.

From 1998 through 2005, a total of 23,216 pulmonary NTM–associated hospitalizations were identified, of which 16,475 (71%) were non-AIDS related. Of these, 9,439 (57%) were women and 8,997 (55%) were among persons >70 years of age. The proportion of pulmonary NTM hospitalizations among persons >70 years of age varied by sex: 45% of men and 62% of women were >70 years of age. For both sexes, the average annual prevalence of non-AIDS pulmonary NTM-associated hospitalizations increased with age, but among persons >70 years of age, the relative prevalence was higher for women than for men. The relative prevalence for persons 70–79 years of age compared with those 40–49 years of age was 15-fold higher for women (9.4/100,000 vs. 0.6/100,000), and 9-fold higher for men (7.6/100,000 vs. 0.8/100,000); similar relative differences were seen in the >80–95-year age group (Figure 1).

**Figure 2:**
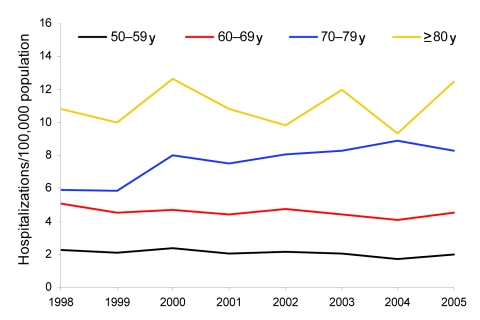
Prevalence of non-AIDS pulmonary nontuberculous mycobacteria–associated hospitalizations among men by age group and year, Healthcare Cost and Utilization Project (HCUP) state inpatient databases, USA, 1998–2005.

**Figure 3:**
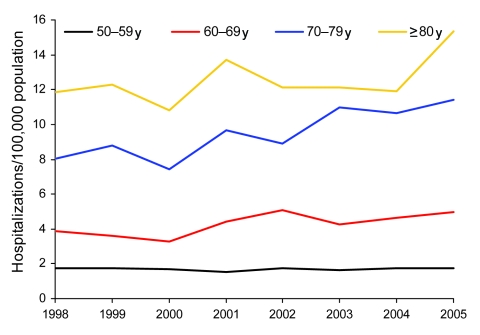
Prevalence of non-AIDS pulmonary nontuberculous mycobacteria–associated hospitalizations among women by age group and year, Healthcare Cost and Utilization Project (HCUP) state inpatient databases, USA, 1998–2005.

To study trends within the older age groups over time, we restricted our analysis to the >50-year age group and examined trends during the period 1998–2005 for men and women separately. Among men, the prevalence decreased significantly among the 50–59-year age group (2.7% per year; p = 0.011 by χ^2^ test), and increased significantly among men 70–79 years of age (5.3% per year; p = 0.0001 by χ^2^ test); no significant changes were evident in the other age groups (Table 1; Figure 2). Among women, the prevalence increased significantly for women 60–79 years of age with an average annual increase of 4.6% (p = 0.0069 by χ^2^ test) among women 60–69 years of age and 5.5% (p<0.0001) among women 70–79 years of age (Table 2; Figure 3).

**Table 1:** Results of Poisson regression modeling for trends in pulmonary NTM, HCUP-SID, USA, 1998–2005*

Group	Sex	Annual % change	Wald 95% CI	p value
State				
California	M	–1.5	–4.0–1.3	0.24
	F	–1.5	–3.3–0.3	0.10
New York	M	–2.7	–5.9–0.54	0.10
	F	4.5	1.1–8.2	0.0097
Florida	M	3.2	0.76–5.7	0.010
	F	6.3	3.1–9.9	0.00010
Age group, y				
50–59	M	–2.7	–4.8 to –0.61	0.0118
	F	0.14	–1.4–1.7	0.8629
60–69	M	–1.5	–3.1–0.01	0.0490
	F	4.6	1.2–8.0	0.0069
70–79	M	5.3	2.5–8.2	0.0001
	F	5.5	2.9–8.2	<0.0001
>80	M	0.65	–3.0–4.4	0.7327
	F	2.5	–0.62–5.7	0.1177

*NTM, nontuberculous mycobacteria; HCUP, Healthcare Cost and Utilization Project; SID, state inpatient databases; CI, confidence interval. The scaled Pearson χ^2^ (value/df) was 1 for all items.

**Table 2:** Primary diagnoses, non-AIDS pulmonary NTM-associated hospitalizations, HCUP-SID, USA, 1998–2005*

ICD-9 code	Primary diagnosis	No. (%)
0310	Pulmonary NTM	5,148 (31.25)
482	Pneumonia	1,156 (7.01)
49121	Obstructive chronic bronchitis with acute exacerbation	821 (4.98)
51881	Acute respiratory failure	392 (2.38)
4280	Congestive heart failure, unspecified	225 (1.37)
4941	Bronchiectasis with acute exacerbation	216 (1.31)
2765	Volume depletion	196 (1.19)
515	Postinflammatory pulmonary fibrosis	186 (1.13)
5070	Aspiration pneumonia caused by inhalation of food/vomitus	176 (1.07)
	Other primary diagnosis <1% of population	7,959 (48.3)

*NTM, nontuberculous mycobacteria; HCUP, Healthcare Cost and Utilization Project; SID, state inpatient databases; ICD-9, International Statistical Classification of Diseases, Revision 9.

**Figure 4:**
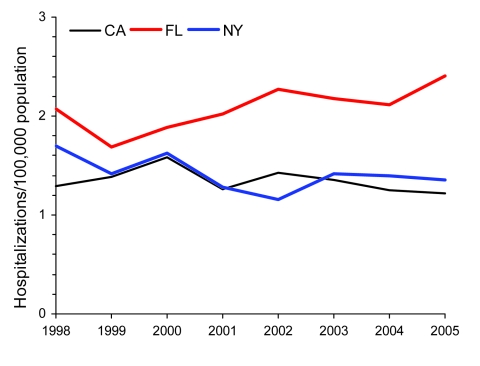
Age-adjusted prevalence of non-AIDS pulmonary nontuberculous mycobacteria–associated hospitalizations among men, California (CA), Florida (FL), and New York (NY), USA, Healthcare Cost and Utilization Project state inpatient databases, 1998–2005.

**Figure 5:**
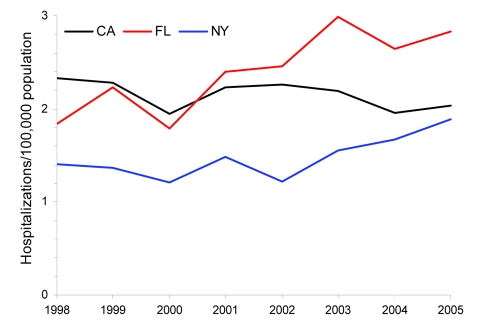
Age-adjusted prevalence of non-AIDS pulmonary nontuberculous mycobacteria–associated hospitalizations among women, California (CA), Florida (FL), and New York (NY), USA, Healthcare Cost and Utilization Project state inpatient databases, 1998–2005.

We studied trends by geographic area and chose the 3 states with the greatest numbers of annual observations during the study period to ensure robust trend analysis. California, Florida, and New York represent unique regions in the United States and overall comprised 62% of NTM hospitalizations in the 11 states included in the analysis. To compare prevalence across these states, we calculated age-adjusted prevalence for men and women. Among both sexes, prevalence was highest in Florida; a significant annual increase was seen from 1998 through 2005. Among men, the average annual age-adjusted prevalence in Florida was 2.1/100,000 population, with a significant increase from 2.1 to 2.4 (3.2% increase/year); the average annual prevalence in California was 1.3 and for New York 1.4, with no significant change during the study period (Table 1, Figure 4). Among women, the average annual age-adjusted prevalence in Florida was 2.4/100,000; an increase of 1.8 in 1998 to 2.8 in 2005 (average 6.5%/year) was identified. For women in New York, annual prevalence increased significantly from 1.4/100,000 to 1.9/100,000 (4.6%/year); no significant change was detected in California (Table 1; Figure 5.)

Among the 16,475 non-AIDS pulmonary NTM–associated hospitalizations during 1998–2005, a total of 5,148 (31%) hospitalizations had pulmonary NTM as a primary diagnosis. The other leading primary diagnoses were pneumonia (7%), obstructive chronic bronchitis with acute exacerbation (5%), acute respiratory failure (2%), congestive heart failure (1.4%), and bronchiectasis (1.3%). No other single primary diagnosis comprised >1% of the primary diagnosis (Table 1). We analyzed secondary diagnoses to identify associated underlying illnesses for hospitalizations where pulmonary NTM was the primary diagnosis. Hospitalizations could be associated with combinations of up to 29 secondary diagnoses, such that the sum of the underlying illnesses identified in any of those fields could add up to >100%. Of these, preexisting cardiovascular conditions, such as hypertension and atrial fibrillation, were most common (47%). Structural lung diseases, such as COPD (34%) and bronchiectasis (15%), were also common (Table 3).

**Table 3:** Secondary diagnoses in hospitalizations in which non-AIDS pulmonary NTM is the primary diagnosis, HCUP-SID, USA, 1998–2005*

Secondary diagnosis	Total no.	% Pulmonary NTM as primary diagnosis
Cardiovascular conditions	2,441	47.4
COPD	1,724	33.5
Nutrition/hydration conditions	1,396	27.1
Bronchiectasis	769	14.9
Anemia	536	10.4
Pneumonia	467	9.1
Hemoptysis	438	8.5
Endocrine disorders	393	7.6
Postinflammatory pulmonary fibrosis	388	7.5
Esophageal reflux	295	5.7
Acute respiratory failure	184	3.6

*NTM, nontuberculous mycobacteria; HCUP, Healthcare Cost and Utilization Project; SID, state inpatient databases; COPD, chronic obstructive pulmonary disease.

**Figure 6:**
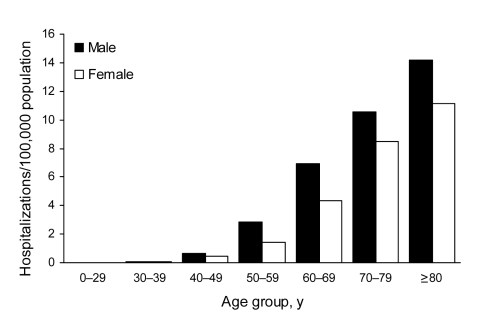
Prevalence of chronic obstructive pulmonary disease as a secondary diagnosis by age group and sex when non-AIDS pulmonary nontuberculous mycobacteria is the primary diagnosis, Healthcare Cost and Utilization Project state inpatient databases, USA, 1998–2005.

**Figure 7:**
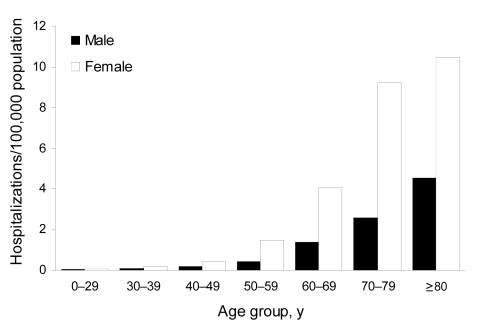
Prevalence of bronchiectasis as a secondary diagnosis by age group and sex when non-AIDS pulmonary nontuberculous mycobacteria is the primary diagnosis, Healthcare Cost and Utilization Project state inpatient databases, USA, 1998–2005.

To identify distinct patterns of underlying illnesses by sex, we analyzed the age and sex distribution for selected underlying illnesses among hospitalizations where non-AIDS pulmonary NTM was the primary diagnosis. For hospitalizations with secondary diagnoses related to COPD, the prevalence of hospitalization was higher for men than for women in all age groups, ranging from 2-fold in the 50–59-year age group to 1.3× greater in the >70-year age group (Figure 6). Among persons hospitalized with bronchiectasis as a secondary diagnosis, the prevalence was consistently higher in women than in men in all age groups, ranging from 3-fold higher in the 50–59-year age group to 4-fold in the 70–79-year age group (Figure 7).

## Discussion

We present nationally representative population-based prevalence estimates for pulmonary NTM disease, age-specific prevalence estimates for the United States, and prevalence data available on hospitalizations associated with pulmonary NTM disease. Estimates of this type were reported in 1987 (*10*). In addition, we demonstrate an increasing prevalence of pulmonary NTM-associated hospitalizations among both men and women in Florida, different than that for California and New York, and identify regional differences in disease activity as has been previously suggested (*19*).

The increased prevalence among those >50 years of age indicates a disease process with onset in the fifth or sixth decade of life, either as a result of an underlying genetic susceptibility or onset of underlying illnesses (e.g., COPD). Although our data are derived from hospitalizations associated with NTM rather than outpatient visits, which might be more likely to occur earlier in the disease course, data from outpatient settings show a similarly increased disease effect in the >50 year-old population (*1**,**3**,**5*). Because prevalence is a function of disease incidence and duration, the highest prevalence in the oldest age groups likely reflects new cases as well as the accumulation of existing cases, i.e., persons living with the disease. For this reason we cannot draw more specific conclusions regarding age at onset of illness.

Among persons >70 years of age, the higher age-specific prevalence of women relative to men is consistent with prior single site studies showing a predominance of pulmonary NTM diagnosed in women (*1**,**3**–**5*), an apparent change from the 1970s and 1980s when men predominated among cases of pulmonary NTM (*10*). Although women aged >70 years have an increased prevalence relative to men in the same age group, the effect among men is still substantial. NTM in women may predominate in more recent clinical studies because women outnumber men in the older age groups; when number of cases relative to their representation in the population are considered (e.g., age-specific disease prevalence), the sex differences are reduced.

The absence of a predominant co-illness is noteworthy, especially in this hospitalized population, and supports the possibility of diverse etiologies for NTM disease. Other than pulmonary NTM, no single diagnosis comprised more than 7% of primary diagnoses. This finding is consistent with observations from recent single-site studies of an increasing proportion of cases having no known risk factors, particularly among women (*1**,**3**–**5*). Bronchiectasis, a defining feature for NTM disease (*20*), was identified and coded as the primary diagnosis in only 1.3% of hospitalizations caused by NTM. Among discharged patients for whom pulmonary NTM was the primary diagnosis, 15% had bronchiectasis listed as a secondary diagnosis. Because the criteria for defining NTM disease include bronchiectasis, we suspect that a higher proportion of patients than were reported actually had this condition.

The reasons for the low proportion are unclear, but may reflect the relative difficulty of diagnosing bronchiectasis without a computed tomography scan. In this same group having NTM as a primary discharge diagnosis, 47% had cardiac conditions and 33% had COPD/emphysema. The more frequent diagnosis of COPD among men having pulmonary NTM and of bronchiectasis among women with pulmonary NTM is consistent with previous studies (*3**,**21**,**22*). Although some of this difference in disease presentation could be related to a gender diagnostic bias (*23*), it may also be related to a number of biologic factors encompassing genetic, immunologic (*24**,**25*), and anatomic cofactors. Hormonally mediated sex-based responses to inflammation have been postulated as a pathophysiologic mechanism (*23**,**26*) for pulmonary NTM disease. Even among persons with cystic fibrosis, who have a well characterized genetic predisposition to pulmonary NTM disease, sex differences exist (*23**,**27*). Finally, a predisposing morphotype of tall, thin white women with underlying illnesses of mitral valve prolapse, scoliosis, and pectus excavatum suggests genetic components to the phenotype (*5**,**28*).

The overlap between bronchiectasis and pulmonary NTM is extensive but of unclear etiology. Like pulmonary NTM, bronchiectasis is thought to be a common final manifestation of several conditions, including infectious causes as triggers of inflammation (*22*). Current estimates of bronchiectasis are limited, but a recent analysis of a nationally representative nonhospitalized population estimated a prevalence of 272/100,000 persons >75 years of age in 2001; age and sex distribution was strikingly similar to that for pulmonary NTM (*29*). How much of bronchiectasis represents undiagnosed NTM-associated disease is unclear. Among persons >65 years of age in the United States, 26% of patients with chronic heart failure also had COPD and bronchiectasis, and these conditions posed an increased risk for hospitalization (*30*).

The regional differences in prevalence and trends of pulmonary NTM hospitalizations are intriguing. *Mycobacterium avium* complex, the most common group of NTM causing infection in humans, can be acquired through exposure to either soil or water. Whether these geographic differences in prevalence are caused by differential exposure to NTM in certain regions related to human activity or to increased concentrations of mycobacteria in certain environments, or both, is not clear. Heterogeneity in geographic prevalence of disease, NTM isolation, and mycobacterial growth has been demonstrated previously; some of the highest disease and isolation prevalence are found in the southeastern United States, particularly along the coastal regions of the Atlantic and Gulf coasts. A higher prevalence of NTM exposures in these areas, based on skin hypersensitivity tests, was first demonstrated in surveys of Navy recruits using purified protein derivative B (*M. intracellulare*) (*19*).

Subsequent surveys of NTM isolates on the basis of patient isolates referred to state public health laboratories found a greatly elevated prevalence of isolation in Florida (29/100,000 population), relative to California (1.7/100,000 population) and New York (2.0/100,000 population) (*31*). More recently, a multisite study of pulmonary NTM prevalence among cystic fibrosis patients found the highest prevalence primarily at sites in the southeastern and southwestern coastal areas (*32*). Higher average temperature and humidity in these areas could favor mycobacterial growth or survival in aerosol droplets. NTM have been isolated and identified in drinking water systems throughout the United States, including those with a variety of water sources (surface/groundwater), water types (hard/soft; high/low organic), and disinfectants used (chlorine/ozone) (*33**,**34*). The acidic, brown water swamps in the southeastern United States, particularly along the coastal region of the Atlantic and Gulf shores, harbor high numbers of NTM. DNA fingerprinting techniques applied to NTM isolates have shown the identical pattern among isolates obtained from patients and their drinking water supply (*35**,**36*). Many NTM species have high innate chlorine and biocide resistance, and therefore treatment of municipal water systems with these disinfecting agents may shift the bacterial population towards mycobacteria. Furthermore, some of these species can persist in flowing water distribution systems through their creation of biofilms (*37*).

This study had several limitations. First, these data represent a hospitalized population; most pulmonary NTM diseases are diagnosed and managed in the outpatient setting. Prevalence trends are likely to be different in outpatient populations, depending on the factors influencing hospitalization. Because persons may be more likely to be hospitalized later in the course of the disease, our data could therefore be skewed toward an older population. In a recent case-series of nonhospitalized patients (95% women), the average age at diagnosis was 56 years (*5*). In our study, women >70 years of age predominated. However, until we have better data on outpatients, we cannot definitively know the nature and direction of this bias. Although the populations of some states included in this analysis may have a higher proportion of elderly persons, we accounted for this by estimating age-adjusted or age-specific prevalence.

We cannot know from these data whether the trends in Florida are due to immigration of retirees from other areas, however, geographic differences in exposure have been noted among young Navy recruits who were lifelong residents in their states (*19*). Thus, these differences are unlikely to be explained solely by migration. Awareness of NTM disease may have increased in recent years because of the discovery of new species. Whether this discovery has led to more testing and more frequent diagnosis of NTM along with increased use of commercial molecular probes for the most common species, is uncertain. Also, it is unclear as to whether use of these probes would vary greatly by geographic area. Another limitation is that the validity of the ICD-9-CM codes for NTM is unknown. Because pulmonary NTM is a relatively rare condition, hospitalizations identified by use of these codes likely represent an underestimate of the impact of pulmonary NTM. Because we could not identify multiple hospitalizations for any 1 patient, any given patient could be represented more than once in a given year. However, considering the rarity of this disease it is unlikely that this issue would result in a substantial overestimate of the true impact of pulmonary NTM.

In summary, pulmonary NTM represents an increasing cause of illness in the United States, particularly among women in selected areas. Further research is needed to define the prevalence of disease in nonhospitalized persons in regions throughout the United States and to elucidate risk factors for disease susceptibility as well as environmental exposure.
